# New Regional Species Records for the Moroccan Bee Fauna (Hymenoptera, Apoidea), with a Special Focus on the Marrakesh-Safi Region

**DOI:** 10.3390/insects16090873

**Published:** 2025-08-22

**Authors:** Ayyoub Skaou, Abdessamad Aglagane, Omar Er-Rguibi, Houda Benkhalifa, Ahlam Sentil, Patrick Lhomme, Denis Michez, El Hassan El Mouden

**Affiliations:** 1 Laboratory of Water Science, Microbial Biotechnology and Sustainability of Natural Resources, Faculty of Science Semlalia, Cadi Ayyad University, Marrakech 40000, Morocco; skaouayoub@gmail.com (A.S.); houdaabenkhalifa@gmail.com (H.B.); elmouden@uca.ac.ma (E.H.E.M.); 2 Higher Institute of Nursing Professions and Health Technics, Laayoune 70000, Morocco; omar.er.rguibi@gmail.com; 3 Laboratory of Zoology, Research Institute for Biosciences, University of Mons, 7000 Mons, Belgium; ahlam.sentil@umons.ac.be (A.S.); patrick_lhomme@hotmail.fr (P.L.); denis.michez@umons.ac.be (D.M.); 4 Plant Ecology Unit, Department of Environment and Plant Protection, National School of Agriculture, Meknes 50001, Morocco

**Keywords:** bee distribution, Moroccan bee fauna, new records, wild bees, solitary bees

## Abstract

Although Morocco is a biodiversity hotspot for wild bees, much of its bee fauna remains underexplored. This study aims to enhance knowledge on the regional diversity of Moroccan bees by monitoring wild bee species across nine regions during the 2022–2024 period. A total of 245 bee species—representing 34 genera and 6 families—are documented, accounting for ~25% of the country’s bee species, including 74 species newly recorded at the regional level. We recorded 42 species as new for the Marrakech-Safi region, bringing the total to 597 species and confirming this region as a biodiversity hotspot. New records were also observed in the southern and desert regions, such as Laayoune-Boujdour-Sakia El Hamra and Guelmim-Oued Noun (12 species for each), as well as Dakhla-Oued Ed-Dahab (7 species), highlighting the need for further exploration in these under-sampled areas. The genera *Andrena*, *Lasioglossum*, and *Eucera* were the most diverse, collectively representing 61% of the newly recorded species documented in this study. This research provides valuable insights into the diversity and distribution of Morocco’s bee fauna and calls for standardized monitoring, conservation efforts, and expanded research—particularly in underexplored areas such as the southern regions—to preserve Morocco’s vital bee populations and ensure the sustainability of its agroecosystems.

## Simple Summary

Morocco is recognized as a biodiversity hotspot for wild bees. However, a large proportion of its bee species’ regional distribution is still poorly studied and documented. To fill this gap, a 3-year monitoring program (2022–2024) was undertaken across nine regions of Morocco. This monitoring resulted in documenting 245 species (representing ~25% of Morocco’s known bee fauna) from 6 families and 34 genera. Seventy-four species have been documented as newly recorded at the regional level. Notably, the Marrakesh-Safi region appeared as a national biodiversity hotspot, adding 42 new species to reach a total of 597 species. Understudied southern desert regions—especially Laayoune-Boujdour-Sakia El Hamra (86% new species), Dakhla-Oued Ed-Dahab (78%), and Guelmim-Oued Noun (67%)—revealed exceptionally high proportions of new records, underscoring critical sampling gaps. Moreover, dominant genera such as *Andrena* (61 species), *Lasioglossum* (31), and *Eucera* (29) collectively accounted for 49% of all species and 61% of new records, reflecting their ecological adaptability as ground-nesting generalist pollinators. These findings highlight Morocco’s rich yet underexplored bee fauna and uncover the need for urgent standardized monitoring, expanded research in southern/desert ecosystems, and targeted conservation to safeguard vital pollinators and ensure agroecosystem sustainability.

## 1. Introduction

Bees form one of the most ecologically and economically significant groups within the order Hymenoptera, comprising nearly 21,000 species described globally [1]. As the most vital group of pollinators [2,3], bees are essential for providing ecosystem services [4]. Notably, they support the sexual reproduction of the majority of domesticated and wild flowering plants [5]. Insect pollination significantly contributes to agricultural production, accounting for 25% of its value in North Africa [6,7], with an estimated economic impact of 1.23 billion USD in Morocco [8]. However, recent studies highlight a global decline in insect populations [9,10], including bee populations [11]. Alarmingly, 9.2% of European bee species are categorized as threatened, while over half (56.7%) remain data deficient, obscuring their conservation status [12]. This alarming trend has largely been attributed to agricultural intensification and climate change [13]. Given the critical role of pollinators in ecosystems, expanding research on bees—particularly in Africa, a region that remains largely under-investigated [14]—is essential to address knowledge gaps related to their distribution and ecology and to implement effective conservation strategies to mitigate the ongoing decline of these vital species.

Morocco stands out as one of the Mediterranean basin’s most biodiverse countries in terms of bees, containing 961 known species listed in the checklist of Lhomme et al. [15], excluding those that have been newly recorded and described [16,17,18,19,20]. Several recent studies focusing on species inventories have been conducted in various Moroccan contexts, including agroecosystems (e.g., [21]), wild and touristic areas (e.g., [22]), as well as environments combining both aspects (e.g., [23]). Additionally, research on the conservation of bees in agricultural systems has also been conducted [24,25,26]. Despite notable studies reporting high species diversity, research on pollinators—and particularly bees—remains underdeveloped when compared to other Mediterranean countries. Therefore, it is crucial to broaden research efforts on bee diversity and geographic distribution across a broader range of environments and regions in Morocco.

Monitoring programs for bees serve a crucial role in tracking biodiversity trends, allowing researchers to assess ecological changes and evaluate the impacts of land management practices [27]. However, the effectiveness of such efforts is severely limited by a persistent lack of detailed, high-quality data on the diversity and spatial distribution of bee species, particularly at the local and national levels [28]. This data deficiency presents a major obstacle to evidence-based conservation planning, making it difficult to detect population trends, highlight species at risk, and prioritize areas for protection [29]. Addressing these knowledge gaps is fundamental to designing robust and long-term strategies to safeguard wild bee populations.

The present study is situated within this context, aiming to enhance our understanding of wild bee diversity in Morocco. Special attention is given to the Marrakech-Safi region, which is recognized as a national hotspot for solitary bees, with 511 species documented to date [15]. The main objectives of this study are presented as follows: (1) to update regional bee checklists and expand the current knowledge of bee diversity across Moroccan regions, with a particular focus on the Marrakech-Safi region; (2) to investigate additional areas that have been undersampled; and (3) to provide foundational data to inform conservation and agroecosystem management in Morocco.

## 2. Materials and Methods

### 2.1. Sampling

**Figure 1:**
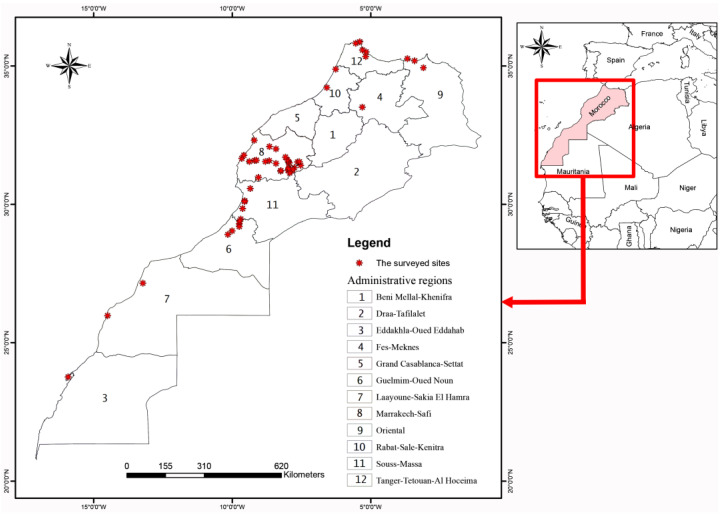
Map of Morocco indicating the sampled sites.

**Figure 2:**
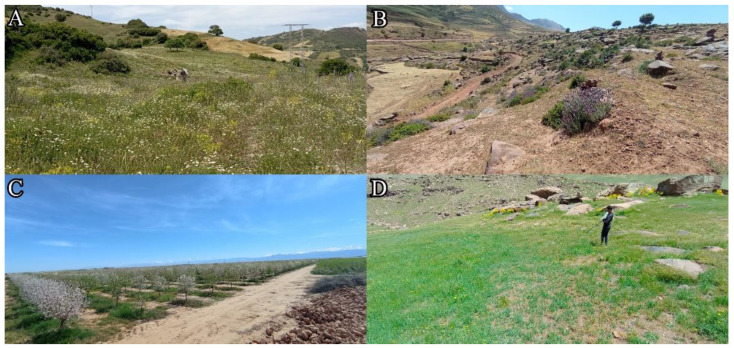
Some sites studied during the monitoring ((**A**) Ksar Sghir, 35.8234, −5.5370; (**B**) Tikhfist, 31.2558, −7.8191; (**C**) Mejjat, 31.4804, −8.4185; (**D**) Oukaïmeden, 31.2136, −7.8509).

The material studied was collected during field trips conducted across 57 distinct sites (Supplementary Table S1), covering 9 out of the 12 Moroccan administrative regions (Figure 1). The sampling was conducted over a 3-year period (2022–2024). The sampling sites represent a typical combination of natural, semi-natural, and agricultural ecosystems (Figure 2). All bee captures occurred between January and July, coinciding with the peak activity period of bees. Most sites in the Marrakech-Safi region were sampled more than twice, whereas sites in other regions were generally sampled once.

During this study, two sampling methods were randomly employed: active sampling using entomological nets and passive collection methods through the use of colored pan traps (blue, white, and yellow), which were employed as a complementary approach. Active sampling using entomological nets was performed at all sites, with approximately 30 min to 1 h of sampling in natural sites and 3 to 4 h in agroecosystems. However, passive sampling was conducted using pan traps only at agricultural sites, with 12 sets of triplets (blue, white, and yellow bowls) deployed per site [30]. Each rectangular bowl (20.2 cm length, 11.2 cm width, and 5.2 cm height) was filled with 400 mL of water and a drop of liquid soap. Pan traps were deployed on the soil surface during each sampling event for a duration of 8 h (9:00 a.m. to 5:00 p.m.).

### 2.2. Bee Identification

All collected bee specimens were first identified at the genus level [31]. Following this preliminary identification, the specimens were sent to expert taxonomists for species-level identification (see acknowledgments for details). The studied specimens were dried, pinned, and carefully stored at the entomological collection of the Faculty of Science Semlalia, Cadi Ayyad University, which now serves as a reference for future research. All specimens have been properly vouchered and made publicly accessible.

### 2.3. Update to the Regional Checklist

The bee species identified in this study were compared with those reported in previous peer-reviewed publications [15,16,17,18,19,20,32,33]. This comparative approach not only confirmed the presence of previously recorded species but also contributed to updating and refining the known regional distribution of the collected bee taxa. By carefully assessing the geographic range of each species, we enhanced the accuracy of existing regional checklists, thus providing valuable new insights into the current distribution patterns of Moroccan bee species.

### 2.4. Examined Material

Information about the examined material is presented in a standardized format to ensure clarity and consistency (Appendix A). Each entry begins with a bullet point, indicating the start of the material citation, followed by the number and sex of the collected specimens. This is followed by the locality name, geographical coordinates, date of collection, altitude (alt.), and the name of the collector (leg.). The method of collection—whether using sweep nets or pan traps (with specific colors: blue, white, or yellow)—is also indicated. In cases where a species was collected from multiple localities, the specimens are organized alphabetically by locality and then by the date of collection within each locality. Additionally, for each newly recorded species, the bee taxonomist (Det) is indicated, and both global and regional distribution data are provided (regions indicated in bold denote new regional records), offering context for its presence in the studied region.

## 3. Results

### 3.1. Bee Species Records

**Figure 3:**
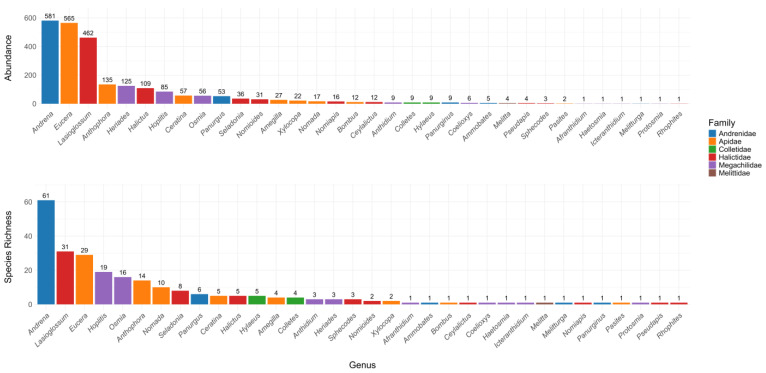
Total bee species abundance and richness of collected bee genera.

This study generated a total of 2467 bee specimens belonging to 245 species, which are distributed across 34 genera and 6 families (Table 1). The Apidae family, represented by eight genera, was the most abundant (837 specimens), accounting for 34% of all collected specimens. This is followed by the Halictidae family (674 specimens), represented by nine genera and accounting for 27% of the total specimens. The Andrenidae family ranked third, with 649 specimens distributed across five genera, accounting for 26% of the total specimens. The Megachilidae family, with 285 specimens, was represented by nine genera and accounted for 12% of the total captured bees. The two remaining families represented less than 1% of the specimens collected (Figure 3). In terms of species richness, Andrenidae and Apidae lead with 70 (29%) and 66 species (27%), respectively. These are followed by Halictidae with 53 species (22%), Megachilidae with 46 species (19%), Colletidae with 9 species (4%), and Melittidae with 1 species (0.5%) (Figure 3). The most species-rich genera were *Andrena* (61 species), representing 25% of all collected species, followed by *Lasioglossum* (31 species), *Eucera* (29 species), *Hoplitis* (19 species), *Osmia* (16 species), *Anthophora* (14 species), and *Nomada* (10 species). All other recorded genera displayed species richness values below 10 (Figure 3).

### 3.2. Newly Recorded Species

**Figure 4:**
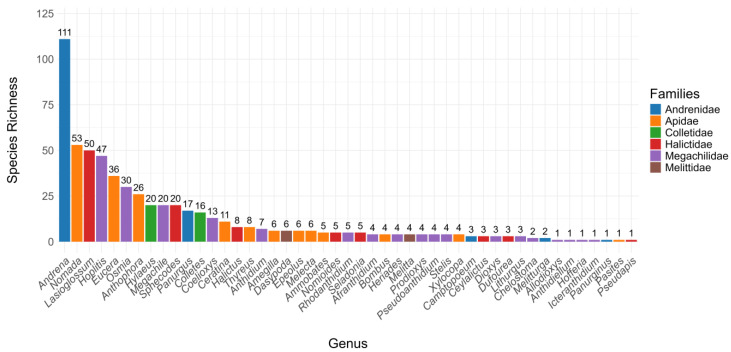
Species richness of the revised checklist of bee genera recorded in the Marrakech-Safi region.

Appendix A provides an overview of the new regional species records (NRSRs) identified in each region, with further details outlined below. A total of 74 NRSRs were documented across eight regions during this study, spanning 6 families and 21 genera. Among these, the following three genera were particularly dominant: *Andrena* with 19 NRSRs, *Eucera* with 17 NRSRs, and *Lasioglossum* with 10 NRSRs. The Marrakech-Safi region is where we observed the highest number of new records, accounting for 42 NRSRs. This was followed by Laayoune-Boujdour-Sakia El Hamra and Guelmim-Oued Noun (12 NRSRs each), Tanger-Tetouan-Al Hoceima (9 NRSRs), Dakhla-Oued Ed-Dahab (7 NRSRs), the Oriental region (2 NRSRs), and Souss-Massa and Rabat-Sale-Kenitra (1 NRSR each). Interestingly, Laayoune-Boujdour-Sakia El Hamra exhibited the highest proportion of NRSRs, with 86% of the total collected species being new to the region. Other regions also demonstrated notable rates of newly recorded species: 78% in Dakhla-Oued Ed-Dahab, 67% in Guelmim-Oued Noun, 50% in the Oriental region, 32% in Tanger-Tetouan-Al Hoceima, 20% in Marrakech-Safi, 14% in Rabat-Sale-Kenitra, and 7% in Souss-Massa (Supplementary Table S2). The updated bee fauna checklist for the Marrakech-Safi region is now 597 species (Figure 4 and Supplementary Table S3), including the results from recent studies (i.e., 41 new species) and the present study (Table 2).

## 4. Discussion

The monograph by Lhomme et al. [15] represents a significant milestone in advancing the understanding of Moroccan bee fauna, serving as a foundational reference for researchers interested in pollinator diversity in the region. Since its publication, numerous studies have confirmed the remarkable diversity of bees across various ecosystems, while simultaneously underscoring a critical gap in research related to the Moroccan bee fauna by indicating that despite its ecological and economic importance, it remains significantly underexplored when compared to other regions in the Mediterranean basin [17,18,19,20,34,35].

The present study has built upon these foundational findings to further investigate the diversity of wild bees across nine Moroccan regions, providing a significant contribution to our understanding of this critical group of pollinators. Over a 3-year period, we succeeded in capturing a diverse array of species distributed across Morocco. In total, 245 bee species representing 34 genera and 6 families were documented, including 74 NRSRs that mark important additions to the available regional records. The total number of bee species collected during this study represents approximately 25% of all species found in Morocco [15].

The findings confirm the Marrakech-Safi region as a key hotspot for bee diversity, with 42 newly recorded species bringing the total to 597 species. This makes the region the most diverse in the country in terms of bee species [15]. This high species richness is likely driven by a combination of ecological and methodological factors. On one hand, this result highlights the region’s ecological significance, particularly due to the diversity and heterogeneity of habitats—from lowland agricultural plains to mountain grasslands—and the altitudinal gradient associated with the High Atlas Mountains [15]. The region also benefits from diverse floral resources provided by both native vegetation and cultivated crops, which can sustain large and varied bee communities throughout the year. This creates heterogeneity in floral phenology across habitats, which facilitates the coexistence of species with different foraging preferences. On the other hand, given its status as a touristic area, this region’s high diversity could also be influenced by an oversampling bias, commonly known as the Wallacean Shortfall, where increased sampling efforts in accessible and frequently visited areas contribute to an overrepresentation of biodiversity [36]. Consequently, while the recorded species richness highlights the region’s ecological value, it is also essential to consider potential sampling biases that may overestimate the true extent of bee diversity in the region.

Interestingly, despite being considered one of the most well-studied areas for bee fauna, nearly 20% of the collected species in Marrakech-Safi represented new records, emphasizing the ongoing need for further exploration and highlighting that even intensively studied areas can harbor undocumented diversity. Additionally, a significant proportion of these new records were observed in southern regions, such as Laayoune-Boujdour-Sakia El Hamra, Dakhla-Oued Ed-Dahab, and Guelmim-Oued Noun, where 86, 78, and 67% of the collected species, respectively, have never been reported. According to the national checklist, these regions previously accounted for only 17, 6, and 140 species, respectively [15]. These findings further confirm that the relatively low species richness reported in these areas is more reflective of limited sampling effort rather than an actual lack of bee diversity [15]. These results not only reveal the remarkable richness of Morocco’s bee fauna but also draw attention to regions that remain significantly underexplored. They also highlight an urgent need for continued research, particularly in desertic regions, which are likely to host unique and potentially endemic species [15]. Notably, our study was seasonally limited, which may underrepresent bee species that are active in late summer and fall. Thus, it is essential to extend sampling efforts both geographically and temporally (i.e., throughout the year) to achieve a more comprehensive assessment of wild bee diversity and community dynamics, which can inform the implementation of effective and sustainable conservation strategies.

The current study highlights *Andrena* as the most diverse genus, with a total of 61 recorded species. This finding is consistent with recent research conducted in the Marrakech-Safi region, where *Andrena* was also found to be the genus with the highest species richness [19]. This result is rather unsurprising, given that *Andrena* is widely recognized for its exceptional diversity across the Western Palearctic region, where it flourishes in various habitats and environmental conditions [32]. In Morocco, specifically, *Andrena* stands out as the most species-rich genus, with a remarkable total of 202 documented species [15,18,37].

Following *Andrena*, the genera *Lasioglossum* and *Eucera* also demonstrated notable diversity, with 31 and 29 species recorded, respectively. These two genera are well-represented in Morocco’s bee fauna, with *Lasioglossum* comprising 65 species and *Eucera* comprising 54 species in the national checklist [15]. Together, these three genera accounted for nearly half of all species collected in this study (49%). This result is consistent with broader trends across Morocco, where these three genera collectively represent 31.11% of all known bee species [15]. The majority of the newly recorded species collected in this study (61%) belong to three genera: *Andrena* (19 species), *Eucera* (17 species), and *Lasioglossum* (10 species). The dominance of these three genera can be attributed to their ecological adaptability. These are primarily ground-nesting bees and opportunistic in their host plant choices. They can be found in high abundance within agroecosystems on crops such as faba bean, coriander, sweet pea, apple, canola, zucchini, Armenian cucumber, and melon [21].

Despite the high economic value of crop pollination and the crucial role of bees in ecosystem functioning [5,8,38], relevant knowledge in the Moroccan context remains largely insufficient. As pointed out by several authors in recent years [15,17,19], this gap constitutes a major obstacle to the development of conservation strategies, especially as anthropogenic pressures continue to intensify. Furthermore, given that monitoring studies serve a crucial role in pollinator management by providing essential baseline data on species diversity, abundance, and distribution across different landscapes [39], they thus contribute to the development of red lists and IUCN species assessments [37]. Moreover, the presented studies help to identify key pollinator species, track population trends, and detect shifts in community composition caused by agricultural intensification or climate change. By highlighting biodiversity hotspots and regions facing ecological stress, inventories guide targeted conservation efforts and inform land-use planning. Therefore, it is crucial to strengthen research efforts to fill scientific gaps and provide a solid knowledge base of Moroccan bees. Simultaneously, it is equally important to raise awareness among farmers regarding the significance of these bees and the need to adopt agricultural practices that are favorable to pollinators. An integrated approach, combining in-depth scientific research and targeted awareness campaigns for farmers, is essential to ensure the sustainability of the ecosystem services provided by bees within the framework of sustainable and resilient agriculture.

## 5. Conclusions

This study provides valuable insights into the diversity of Morocco’s bee fauna, significantly contributing to the conservation of these essential pollinators and the sustainability of agroecosystems in the country. By sampling across nine Moroccan regions, this study has documented a total of 245 bee species, including 74 NRSRs. Notably, the Marrakech-Safi region is highlighted as a major hotspot for bee diversity, with 42 new species recorded, bringing the total to 597 species. Furthermore, southern regions such as Laayoune-Boujdour-Sakia El Hamra, Dakhla-Oued Ed-Dahab, and Guelmim-Oued Noun, which were previously undersampled, revealed significant proportions of new records, emphasizing the need for further research in these areas. This study highlights the need for national monitoring programs and research initiatives on bee diversity, biogeography, and ecology to inform effective and sustainable conservation strategies.

## Figures and Tables

**Table 1:** Bee genera recorded in this study, with species richness (i.e., number of distinct bee species per genus) and abundance (i.e., the number of bee individuals collected per genus) indicated.

Family	Genus	Species Richness	Abundance
Andrenidae	*Ammobates*	1	5
Andrenidae	*Andrena*	61	581
Andrenidae	*Melitturga*	1	1
Andrenidae	*Panurginus*	1	9
Andrenidae	*Panurgus*	6	53
Apidae	*Amegilla*	4	27
Apidae	*Anthophora*	14	135
Apidae	*Bombus*	1	12
Apidae	*Ceratina*	5	57
Apidae	*Eucera*	29	565
Apidae	*Nomada*	10	17
Apidae	*Pasites*	1	2
Apidae	*Xylocopa*	2	22
Colletidae	*Colletes*	4	9
Colletidae	*Hylaeus*	5	9
Halictidae	*Ceylalictus*	1	12
Halictidae	*Halictus*	5	109
Halictidae	*Lasioglossum*	31	462
Halictidae	*Nomiapis*	1	16
Halictidae	*Nomioides*	2	31
Halictidae	*Pseudapis*	1	4
Halictidae	*Rhophites*	1	1
Halictidae	*Seladonia*	8	36
Halictidae	*Sphecodes*	3	3
Megachilidae	*Afranthidium*	1	1
Megachilidae	*Anthidium*	3	9
Megachilidae	*Coelioxys*	1	6
Megachilidae	*Haetosmia*	1	1
Megachilidae	*Heriades*	3	125
Megachilidae	*Hoplitis*	19	85
Megachilidae	*Icteranthidium*	1	1
Megachilidae	*Osmia*	16	56
Megachilidae	*Protosmia*	1	1
Melittidae	*Melitta*	1	4
Total	34	245	2467

**Table 2:** Newly recorded bee genera in the Marrakech-Safi region after the published checklist in 2020, with bee species richness (i.e., number of distinct bee species per genus) indicated.

	Bee Species Richness		
Genus	Present Study ^1^	Revised Publications ^2^	Total
*Ammobates*	-	1	1
*Amegilla*	1	-	1
*Andrena*	14	12	24
*Anthophora*	3	4	7
*Chelostoma*	-	1	1
*Coelioxys*	-	1	1
*Eucera*	8	4	12
*Hoplitis*	1	6	7
*Lasioglossum*	9	2	11
*Megachile*	-	6	6
*Osmia*	1	3	4
*Rophites*	-	1	1
*Tetralonia*	-	1	1
*Thyreus*	-	2	2
*Ceratina*	2	-	2
*Nomiapis*	1	-	1
*Seladonia*	1	-	1
*Sphecodes*	1	-	1
Total newly recorded species	42	44	86
Total recorded by Lhomme et al. [15]			511
Total			597

^1^ New species record reported in the present study. ^2^ New species record reported in revised publications after Lhomme et al. [15].

## Data Availability

The original contributions presented in this study are included in the article/Supplementary Materials. Further inquiries can be directed to the corresponding author.

## Supplementary Materials

The following supporting information can be downloaded at: https://www.mdpi.com/article/10.3390/insects16090873/s1, Table S1: Sites where bee sampling took place; Table S2: Total collected bee species abundance and richness, with new regional species records; Table S3: Revised checklist of bee species recorded in the Marrakech-Safi region.

## Appendix A

**Table A1:** New regional species records (organized alphabetically by family, genus, and species) and their regions of occurrence (abbreviations: OL, Dakhla-Oued Ed-Dahab; GN, Guelmim-Oued Noun; LS, Laayoune-Boujdour-Sakia El Hamra; MS, Marrakech-Safi; OF, Oriental; RK, Rabat-Sale-Kénitra; SS, Souss-Massa; TC, Tanger-Tetouan-Al Hoceima). An X denotes a new record.

Bee Species	MS	TC	SS	RK	LS	GN	OF	OL
*Andrena abscondita*	X							
*Andrena alfkenella*	X							
*Andrena boyerella*		X						
*Andrena djelfensis*	X							
*Andrena discors*	X				X	X		X
*Andrena impunctata*	X					X		
*Andrena innesi*						X		
*Andrena kamarti*								X
*Andrena limata*							X	
*Andrena medeninensis*	X							
*Andrena quieta*	X							
*Andrena rhyssonota*	X							
*Andrena rotundata*	X							
*Andrena rufescens*								X
*Andrena schmiedeknechti*	X							
*Andrena tadorna*	X							
*Andrena varicornis*	X							
*Andrena vulpecula*	X							
*Andrena varia*	X							
*Melitturga caudata*			X					
*Panurginus albopilosus*						X		
*Panurgus maroccanus*								X
*Panurgus pici*		X						X
*Panurgus rungsii*								X
*Amegilla velocissima*	X							
*Anthophora libyphaenica*	X							
*Anthophora romandii*	X							
*Anthophora ventilabris*	X							
*Ceratina chalybea*	X							
*Ceratina citriphila*	X					X		
*Eucera algira*					X			
*Eucera cuniculina*						X		
*Eucera elongatula*		X			X	X		
*Eucera ferruginea*	X							
*Eucera impressiventris*	X							
*Eucera metallescens*	X							
*Eucera nadigi*	X							
*Eucera nigriceps*	X							
*Eucera notata*		X				X		X
*Eucera numida*	X	X						
*Eucera obscura*						X		
*Eucera pumila*	X							
*Eucera strigata*				X				
*Eucera tricincta*							X	
*Eucera vidua*	X					X		
*Eucera warnckei*					X			
*Hylaeus clypearis*						X		
*Ceylalictus variegatus*					X			
*Lasioglossum algericolellum*	X							
*Lasioglossum cristula*		X						
*Lasioglossum interruptum*	X							
*Lasioglossum lucidulum*	X							
*Lasioglossum malachurum*	X							
*Lasioglossum minutissimum*	X							
*Lasioglossum parvulum*	X							
*Lasioglossum pauliani*	X							
*Lasioglossum pauxillum*	X							
*Lasioglossum xanthopus*	X							
*Nomiapis rufiventris*	X							
*Pseudapis nilotica*					X			
*Seladonia cupida*					X			
*Seladonia ochropa*					X			
*Seladonia subaurata*	X							
*Sphecodes rubicundus*	X							
*Anthidium cingulatum*		X						
*Anthidium pullatum*						X		
*Haetosmia circumventa*					X			
*Hoplitis hierichonica*	X							
*Hoplitis mucida*		X						
*Icteranthidium ferrugineum*					X			
*Osmia latreillei*					X			
*Osmia leaiana*		X						
*Osmia melanogaster*	X							
*Melitta schmiedeknecht*					X			

**Examined material**

**Family Andrenidae Latreille, 1802**
**Genus *Andrena* Fabricius, 1775**

***Andrena abscondita* Wood, 2023**
• 1♀; Oukaïmeden; 31.2136, −7.8509; 9 May 2023; alt. 2633 m; A. Skaou leg.; sweep net.
Det: Thomas J. Wood
Global distribution: West-Palearctic
Regional distribution: Fès-Meknès; **Marrakech-Safi**

***Andrena alfkenella* Perkins, 1914**
• 1♀; Asni; 31.2508, −7.9855; 1 April 2022; alt. 1225 m; A. Skaou leg.; sweep net.
Det: Thomas J. Wood
Global distribution: West-Palearctic
Regional distribution: Drâa-Tafilalet; **Marrakech-Safi**

***Andrena boyerella* Dours, 1872**
• 1♂; Fnideq; 35.8763, −5.3941; 25 May 2022; alt. 400 m; A. Skaou leg.; sweep net.
Det: Thomas J. Wood
Global distribution: Near endemic (Morocco, Algeria)
Regional distribution: Béni Mellal-Khénifra; Drâa-Tafilalet; Fès-Meknès; Marrakech-Safi; Souss-Massa; **Tanger-Tétouan-Al Hoceima**

***Andrena discors* Erichson, 1841**
• 5♀; Boujdour; 25.9853, −14.4963; 25 March 2023; alt. 60 m; A. Aglagane and O. Er-rguibi leg.; sweep net. 1♀; Dakhla; 23.7659, −15.9195; 24 March 2023; alt. 5 m; A. Aglagane leg.; sweep net. • 2♀; El Aargoube; 29.4701, −9.6942; 22 March 2023; alt. 22 m; A. Aglagane leg.; sweep net. • 1♀; Guelmim; 29.0411, −10.0072; 23 March 2023; alt. 387 m; A. Aglagane leg.; sweep net. • 3♂; Mejjat; 31.4804, −8.4185; 13 February 2023; alt. 516 m; A. Skaou and O. Er-rguibi leg.; sweep net. • 6♂, 6♀; Mejjat; 31.4804, −8.4185; 20 February 2023; alt. 516 m; A. Skaou and O. Er-rguibi leg.; sweep net. • 3♂; Mejjat; 31.4804, −8.4185; 6 March 2023; alt. 516 m; A. Aglagane leg.; sweep net. • 3♀; Mejjat; 31.4804, −8.4185; 13 March 2023; alt. 516 m; A. Aglagane leg.; sweep net.
Det: Thomas J. Wood
Global distribution: West-Palearctic
Regional distribution: Drâa-Tafilalet; Fès-Meknès; Rabat-Salé-Kénitra; **Dakhla-Oued Ed-Dahab**; **Laâyoune-Boujdour-Sakia El Hamra**; **Marrakech-Safi**; **Guelmim-Oued Noun**

***Andrena djelfensis* Pérez, 1895**
• 1♀; Tikhfist; 31.2558, −7.8191; 20 May 2022; alt. 1780 m; A. Skaou leg.; sweep net.
Det: Thomas J. Wood
Global distribution: West-Palearctic
Regional distribution: Béni Mellal-Khénifra; Fès-Meknès; Oriental; Tanger-Tétouan-Al Hoceima; **Marrakech-Safi**

***Andrena impunctata* Pérez, 1895**
• 6♀; Bouizakarn; 29.2003, −9.7425; 22 March 2023; alt. 837 m; A. Aglagane and O. Er-rguibi leg.; sweep net. • 1♀, 1♂; Mejjat; 31.4804, −8.4185; 13 February 2023; alt. 516 m; A. Aglagane leg.; sweep net. • 3♀; Mejjat; 31.4804, −8.4185; 6 March 2023; alt. 516 m; A. Skaou leg.; sweep net. • 6♀, 2♂; Mejjat; 31.4804, −8.4185; 13 March 2023; alt. 516 m; A. Skaou leg.; sweep net.
Det: Thomas J. Wood
Global distribution: West-Palearctic
Regional distribution: Oriental; **Marrakech-Safi**; **Guelmim-Oued Noun**

***Andrena innesi* Gribodo, 1894**
• 1♂; Bouizakarn; 29.2003, −9.7425; 22 March 2023; alt. 837 m; A. Aglagane leg.; sweep net.
Det: Thomas J. Wood
Global distribution: Palearctic
Regional distribution: Fès-Meknès; **Guelmim-Oued Noun**

***Andrena kamarti* Schmiedeknecht, 1900**
• 1♀; Dakhla; 23.7659, −15.9195; 13 March 2023; alt. 5 m; A. Aglagane leg.; sweep net.
Det: Thomas J. Wood
Global distribution: West-Palearctic
Regional distribution: Fès-Meknès; Rabat-Salé-Kénitra; Tanger-Tétouan-Al Hoceima; **Dakhla-Oued Ed-Dahab**

***Andrena limata* Smith, 1853**
• 1♀; Imahouten; 35.1911, −3.4117; 27 May 2022; alt. 70 m; A. Skaou leg.; sweep net.
Det: Thomas J. Wood
Global distribution: Palearctic
Regional distribution: Drâa-Tafilalet; Fès-Meknès; Marrakech-Safi; Rabat-Salé-Kénitra; **Oriental**

***Andrena medeninensis* Pérez, 1895**
• 1♀; Mejjat; 31.4804, −8.4185; 6 March 2023; alt. 516 m; A. Skaou leg.; white pan trap. • 1♂; Mejjat; 31.4804, −8.4185; 6 March 2023; alt. 516 m; A. Skaou leg.; sweep net. • 1♀; Mejjat; 31.4804, −8.4185; 13 March 2023; alt. 516 m; A. Skaou leg.; sweep net.
Det: Thomas J. Wood
Global distribution: West-Palearctic
Regional distribution: Fès-Meknès; Rabat-Salé-Kénitra; Drâa-Tafilalet; **Marrakech-Safi**

***Andrena quieta* Fabricius, 1775**
• 1♀; Mejjat; 31.4804, −8.4185; 6 March 2023; alt. 516 m; A. Skaou leg.; sweep net.
Det: Thomas J. Wood
Global distribution: Morocco and Tunisia
Regional distribution: Casablanca-Settat; **Marrakech-Safi**

***Andrena rhyssonota* Pérez, 1895**
• 1♀; Oukaïmeden; 31.2136, −7.8509; 12 May 2022; alt. 2633 m; A. Skaou leg.; sweep net. • 1♂; Tikhfist; 31.2558, −7.8199; 12 June 2023; alt. 1788 m; A. Skaou leg.; sweep net.
Det: Thomas J. Wood
Global distribution: West-Palearctic
Regional distribution: Béni Mellal-Khénifra; Casablanca-Settat; Fès-Meknès; Oriental; Rabat-Salé-Kénitra; Tanger-Tétouan-Al Hoceima; **Marrakech-Safi**

***Andrena rotundata* Pérez, 1895**
• 1♂; Ait Ourir; 31.5347, −7.6377; 7 March 2022; alt. 738 m; A. Skaou leg.; sweep net.
Det: Thomas J. Wood
Global distribution: West-Palearctic
Regional distribution: Fès-Meknès; **Marrakech-Safi**

***Andrena rufescens* Pérez, 1895**
• 1♀; Dakhla; 23.7659, −15.9195; 24 March 2023; alt. 5 m; A. Aglagane leg.; sweep net.
Det: Thomas J. Wood
Global distribution: Near endemic (Morocco, Algeria)
Regional distribution: Guelmim-Oued Noun; Marrakech-Safi; Rabat-Salé-Kénitra; Souss-Massa; Tanger-Tétouan-Al Hoceima; **Dakhla-Oued Ed-Dahab**

***Andrena schmiedeknechti* Magretti, 1883**
• 1♀; Asni; 31.2508, −7.9855; 1 April 2022; alt. 1225 m; A. Skaou leg.; sweep net. • 1♂; Asni; 31.2534, −7.9781; 2 April 2022; alt. 1155 m; A. Skaou leg.; sweep net. • 2♂; Douar Bou Azza; 31.5036, −7.9358; 22 March 2022; alt. 358 m; A. Skaou leg.; sweep net. • 1♀, 1♂; Oukaïmeden; 31.2136, −7.8509; 9 May 2023; alt. 2650 m; A. Skaou leg.; sweep net.
Det: Thomas J. Wood
Global distribution: West-Palearctic
Regional distribution: Rabat-Salé-Kénitra; **Marrakech-Safi**

***Andrena tadorna* Warncke, 1974**
• 1♀; Mejjat; 31.4804, −8.4185; 6 March 2023; alt. 516 m; A. Skaou leg.; blue pan trap.
Det: Thomas J. Wood
Global distribution: North Africa
Regional distribution: Oriental; Souss-Massa; **Marrakech-Safi**

***Andrena varia* Pérez, 1895**
• 1♂; Amizmiz; 31.2183, −8.2416; 13 April 2022; alt. 965 m; A. Skaou leg.; sweep net.
Det: Thomas J. Wood
Global distribution: Near endemic (Morocco, Algeria, Tunisia)
Regional distribution: Casablanca-Settat; Fès-Meknès; Rabat-Salé-Kénitra; Tanger-Tétouan-Al Hoceima; **Marrakech-Safi**

***Andrena varicornis* Pérez, 1895**
• 2♀, 1♂; Mejjat; 31.4804, −8.4185; 20 February 2023; alt. 516 m; O. Er-rguibi leg.; blue pan trap and sweep net. • 2♂; Mejjat; 31.4804, −8.4185; 6 March 2023; alt. 516 m; A. Skaou leg.; white pan trap and sweep net.
Det: Thomas J. Wood
Global distribution: West-Palearctic
Regional distribution: Oriental; **Marrakech-Safi**

***Andrena vulpecula* Kriechbaumer, 1873**
• 1♀; Oukaïmeden; 31.2136, −7.8509; 12 May 2023; alt. 2650 m; A. Skaou leg.; sweep net.
Det: Thomas J. Wood
Global distribution: West-Palearctic
Regional distribution: Tanger-Tétouan-Al Hoceima; Rabat-Salé-Kénitra; Fès-Meknès; Souss-Massa; **Marrakech-Safi**

**Genus *Melitturga* Latreille, 1809**

***Melitturga caudata* Pérez, 1879**
• 1♂; Amskroud; 30.5762, −9.3491; 20 May 2022; alt. 607 m; A. Aglagane leg.; sweep net.
Det: Thomas J. Wood
Global distribution: West-Palearctic
Regional distribution: Casablanca-Settat; Marrakech-Safi; Tanger-Tétouan-Al Hoceima; **Souss-Massa**

**Genus *Panurginus* Nylander, 1848**

***Panurginus albopilosus* Lucas, 1849**
• 5♂; El Aargoube; 29.4701, −9.6942; 22 March 2023; alt. 589 m; A. Aglagane and O. Er-rguibi leg.; sweep net. • 3♂; Bouizakarne; 29.2003, −9.7425; 22 March 2023; alt. 873 m; A. Aglagane and O. Er-rguibi leg.; sweep net.
Det: Sébastien Patiny
Global distribution: Near endemic (Morocco, Algeria, Spain, Portugal)
Regional distribution: Drâa-Tafilalet; Fès-Meknès; Marrakech-Safi; Rabat-Salé-Kénitra; Souss-Massa; **Guelmim-Oued Noun**

**Genus *Panurgus* Panzer, 1806**

***Panurgus maroccanus* Pérez, 1895**
• 1♂; Dakhla; 23.7659, −15.9195; 24 March 2023; alt. 5 m; A. Aglagane leg.; sweep net.
Det: Sébastien Patiny
Global distribution: Palearctic
Regional distribution: Fès-Meknès; Marrakech-Safi; Souss-Massa; **Dakhla-Oued Ed-Dahab**

***Panurgus pici* Pérez, 1895**
• 1♀; Al Hoceima; 35.2691, −3.6752; 27 May 2022; alt. 41 m; A. Skaou leg.; sweep net. • 2♂; Beni Ferten; 35.3417, −5.1817; 26 May 2022; alt. 60 m; A. Skaou and A. Aglagane leg.; sweep net. • 2♀, 1♂; Dakhla; 23.7659, −15.9195; 24 May 2023; alt. 05 m; A. Aglagane and O. Er-rguibi leg.; sweep net. • 1♀, 3♂; Fnideq; 35.8763, −5.3941; 25 May 2022; alt. 400 m; A. Skaou and A. Aglagane leg.; sweep net. • 1♀, 1♂; Tamernout; 35.5165, −5.1641; 26 May 2022; alt. 99 m; A. Skaou and A. Aglagane leg.; sweep net.
Det: Sébastien Patiny
Global distribution: Near endemic (Morocco, Algeria, Tunisia)
Regional distribution: Oriental; Souss-Massa; **Tanger-Tétouan-Al Hoceima**

***Panurgus rungsii* Benoist, 1937**
• 1♀; Dakhla; 23.7659, −15.9195; 24 March 2023; alt. 5 m; A. Aglagane leg.; sweep net.
Det: Sébastien Patiny
Global distribution: Morocco. ENDEMIC
Regional distribution: Béni Mellal-Khénifra; Casablanca-Settat; Drâa-Tafilalet; Fès-Meknès; Marrakech-Safi; Oriental; Souss-Massa; **Dakhla-Oued Ed-Dahab**

**Family Apidae Linnaeus, 1758**
**Genus *Amegilla* Linnaeus, 1758**

***Amegilla velocissima* Fedtschenko, 1875**
• 2♀; Ras El Ain; 32.0037, −8.4157; 8 Jun 2024; alt. 486 m; A. Skaou and Y. Dbiba leg.; sweep net.
Det: Pierre Rasmont
Global distribution: Palearctic
Regional distribution: **Marrakech-Safi**

**Genus *Anthophora* Latreille, 1803**

***Anthophora libyphaenica* Gribodo, 1893**
• 1♂; Lagouassem; 31.5316, −7.9647; 22 Mar 2022; alt. 562 m; A. Skaou leg.; sweep net • 1♂; Mejjat; 31.4804, −8.4185; 13 February 2023; alt. 516 m; A. Aglagane leg.; sweep net • 2♂; Oukaïmeden; 31.2136, −7.8509; 13 June 2023; alt. 2650 m; A. Skaou leg.; sweep net.
Det: Pierre Rasmont
Global distribution: West-Palearctic
Regional distribution: Drâa-Tafilalet; Souss-Massa; **Marrakech-Safi**

***Anthophora romandii* Lepeletier, 1841**
• 2♂; Mejjat; 31.4804, −8.4185; 13 February 2023; alt. 516 m; A. Skaou leg.; sweep net • 2♀; Mejjat; 31.4804, −8.4185; 6 March 2023; alt. 516 m; O. Er-rguibi leg.; sweep net • 1♀; Mejjat; 31.4804, −8.4185; 13 March 2023; alt. 516 m; O. Er-rguibi leg.; sweep net.
Det: Pierre Rasmont
Global distribution: West-Palearctic
Regional distribution: Tanger-Tetouan-Al Hoceima; Oriental; Drâa-Tafilalet; **Marrakech-Safi**

***Anthophora ventilabris* Lepeletier, 1841**
• 1♀; Mejjat; 31.4804, −8.4185; 13 February 2023; alt. 516 m; A. Skaou leg.; blue pan trap.
Det: Pierre Rasmont
Global distribution: West-Palearctic
Regional distribution: Béni Mellal-Khénifra; Drâa-Tafilalet; **Marrakech-Safi**

**Genus *Ceratina* Latreille, 1802**

***Ceratina chalybea* Chevrier 1872**
• 1♀; Moulay Brahim; 31.2793, −7.9601; 25 May 2024; alt. 1075 m; A. Skaou leg.; sweep net.
Det: Michael Terzo
Global distribution: Palearctic
Regional distribution: Tanger-Tetouan-Al Hoceima; Oriental; Fès-Meknès; Béni Mellal-Khénifra; Souss-Massa; **Marrakech-Safi**

***Ceratina citriphila* Cockerell, 1935**
• 1♂; Mejjat; 31.4804, −8.4185; 13 February 2023; alt. 516 m; O. Er-rguibi leg.; sweep net. • 1♀; Mejjat; 31.4804, −8.4185; 20 February 2023; alt. 516 m; A. Aglagane leg.; blue pan trap. • 2♂; Mejjat; 31.4804, −8.4185; 6 March 2023; alt. 516 m; A. Skaou and A. Aglagane leg.; white pan trap and sweep net. • 2♀; Mejjat; 31.4804, −8.4185; 13 March 2023; alt. 516 m; A. Skaou leg.; sweep net. • 2♀, 3♂; Mejjat; 31.4804, −8.4185; 30 May 2023; alt. 516 m; A. Skaou leg.; sweep net. • 1♀; Ouaoutelt; 29.2003, −9.7425; 22 March 2023; alt. 480 m; A. Aglagane leg.; sweep net.
Det: Michael Terzo
Global distribution: Palearctic and Afro-Tropical
Regional distribution: Souss-Massa; **Marrakech-Safi**; **Guelmim-Oued Noun**

**Genus *Eucera* Scopoli, 1770**

***Eucera algira* Brullé, 1840**
• 4♂, 1♀; Boujdour; 25.9853, −14.4963; 25 March 2023; alt. 60 m; O. Er-rguibi leg.; sweep net.
Det: Achik Dorchin
Global distribution: West-Palearctic
Regional distribution: Marrakech-Safi; Souss-Massa; Guelmim-Oued Noun; **Laâyoune-Boujdour-Sakia El Hamra**

***Eucera cuniculina* Klug, 1845**
• 1♀; Bouizakarn; 29.2003, −9.7425; 22 March 2023; alt. 837 m; A. Aglagane leg.; sweep net.
Det: Achik Dorchin
Global distribution: West-Palearctic
Regional distribution: Drâa-Tafilalet; **Guelmim-Oued Noun**

***Eucera elongatula* Vachal, 1907**
• 3♂, 2♀; Ahaytouf; 29.3056, −9.7431; 22 March 2023; alt. 1027 m; A. Aglagane leg.; sweep net. • 6♂, 4♀; Bouizakarn; 29.2003, −9.7425; 22 March 2023; alt. 837 m; A. Aglagane leg.; sweep net. • 2♂; Boujdour; 25.9853, −14.4963; 25 March 2023; alt. 60 m; O. Er-rguibi leg.; sweep net. • 4♀; Ksar Sghir; 35.8234, −5.5370; 25 May 2022; alt. 132 m; A. Skaou leg.; sweep net.
Det: Achik Dorchin
Global distribution: West-Palearctic
Regional distribution: Fès-Meknès; Rabat-Salé-Kénitra; Marrakech-Safi; Souss-Massa; Tanger-Tétouan-Al Hoceima; **Laâyoune-Boujdour-Sakia El Hamra**; **Guelmim-Oued Noun**

***Eucera ferruginea* Lepeletier, 1841**
• 1♂; Amizmiz; 31.2044, −8.2466; 13 April 2022; alt. 1129 m; A. Skaou leg.; sweep net. • 1♂; Fnideq; 35.8763, −5.3941; 25 May 2022; alt. 1129 m; A. Skaou leg.; sweep net. • 1♂; Mejjat; 31.4804, −8.4185; 20 February 2023; alt. 516 m; A. Aglagane leg.; sweep net. • 4♂; Oukaïmeden; 31.2136, −7.8509; 13 June 2023; alt. 2560 m; A. Skaou and A. Aglagane leg.; sweep net. • 3♀, 33♂; Tikhfist; 31.2558, −7.8191; 8 May 2023; alt. 1765 m; A. Skaou leg.; sweep net. • 6♀, 3♂; Tikhfist; 31.2558, −7.8166; 12 May 2022; alt. 1765 m; A. Skaou leg.; sweep net. • 2♀, 6♂; Tikhfist; 31.2558, −7.8191; 20 May 2022; alt. 1765 m; A. Skaou, A. Aglagane, and O. Er-rguibi leg.; sweep net. • 1♀; Tikhfist; 31.2558, −7.8212; 12 June 2023; alt. 1765 m; A. Skaou leg.; sweep net.
Det: Achik Dorchin
Global distribution: West-Palearctic
Regional distribution: Fès-Meknès; Drâa-Tafilalet; Souss-Massa; **Marrakech-Safi**; **Tanger-Tétouan-Al Hoceima**

***Eucera impressiventris* Pérez, 1895**
• 2♂; Oukaïmeden; 31.2136, −7.8509; 13 June 2023; alt. 2650 m; A. Skaou and A. Aglagane leg.; sweep net.
Det: Achik Dorchin
Global distribution: West-Palearctic
Regional distribution: Fès-Meknès; Souss-Massa; Guelmim-Oued Noun; **Marrakech-Safi**

***Eucera metallescens* Morawitz, 1888**
• 1♂; Mejjat; 31.4804, −8.4185; 20 February 2023; alt. 516 m; A. Aglagane leg.; yellow pan trap • 2♂; Mejjat; 31.4804, −8.4185; 6 March 2023; alt. 516 m; A. Aglagane leg.; sweep net • 2♀, 1♂; Mejjat; 31.4804, −8.4185; 13 March 2023; alt. 516 m; A. Aglagane and O. Er-rguibi leg.; blue pan trap. • 1♂; Oukaïmeden; 31.2136, −7.8509; 10 June 2022; alt. 2650 m; A. Skaou leg.; sweep net.
Det: Achik Dorchin
Global distribution: West-Palearctic
Regional distribution: Fès-Meknès; Souss-Massa; Guelmim-Oued Noun; **Marrakech-Safi**

***Eucera nadigi* Schulthess, 1924**
• 1♂; Mejjat; 31.4804, −8.4185; 6 March 2023; alt. 516 m; A. Aglagane leg.; sweep net • 2♂; Mejjat; 31.4804, −8.4185; 13 March 2023; alt. 516 m; A. Skaou leg.; sweep net and blue pan trap.
Det: Achik Dorchin
Global distribution: Morocco. ENDEMIC
Regional distribution: Rabat-Salé-Kénitra; **Marrakech-Safi**

***Eucera nigriceps* Morawitz, 1895**
• 1♀; Ouled Hammou; 32.2780, −9.0935; 8 June 2024; alt. 163 m; A. Skaou and Y. Dbiba leg.; sweep net. • 1♂; Ras El Ain; 32.0037, −8.4157; 8 June 2024; alt. 486 m; Y. Dbiba leg.; sweep net.
Det: Achik Dorchin
Global distribution: Palearctic
Regional distribution: Drâa-Tafilalet; **Marrakech-Safi**

***Eucera notata* Lepeletier, 1841**
• 3♀; Al Hoceima; 35.2691, −3.6752; 27 May 2022; alt. 41 m; A. Skaou and A. Aglagane leg.; sweep net. • 2♀; Beni Ferten; 35.8763, −5.3941; 26 May 2022; alt. 60 m; A. Skaou and A. Aglagane leg.; sweep net. • 1♂; Bouizakarn; 29.2003, −9.7425; 22 March 2023; alt. 837 m; A. Aglagane leg.; sweep net. • 2♂; Dakhla; 23.7659, −15.9195; 24 March 2023; alt. 5 m; A. Aglagane leg.; sweep net. • 5♀; Tamernout; 35.5165, −5.1641; 26 May 2022; alt. 1027 m; A. Skaou leg.; sweep net.
Det: Achik Dorchin
Global distribution: West-Palearctic
Regional distribution: Fès-Meknès; Rabat-Salé-Kénitra; Marrakech-Safi; Souss-Massa; **Tanger-Tétouan-Al Hoceima**; **Guelmim-Oued Noun**; **Dakhla-Oued ed-Dahab**

***Eucera numida* Lepeletier, 1841**
• 1♀; Asni; 31.2534, −7.9781; 2 April 2024; alt. 1155 m; A. Skaou leg.; blue pan trap. • 1♀; Fnideq; 35.8763, −5.3941; 25 May 2023; alt. 400 m; A. Skaou leg.; sweep net. • 3♀, 3♂; Mejjat; 31.4804, −8.4185; 13 February 2023; alt. 516 m; A. Skaou, A. Aglagane, and O. Er-rguibi leg.; sweep net • 6♀, 1♂; Mejjat; 31.4804, −8.4185; 20 February 2023; alt. 516 m; A. Skaou, A. Aglagane, and O. Er-rguibi leg.; sweep net • 2♀, 2♂; Mejjat; 31.4804, −8.4185; 6 March 2023; alt. 516 m; A. Aglagane and O. Er-rguibi leg.; sweep net • 1♂; Mejjat; 31.4804, −8.4185; 13 March 2023; alt. 516 m; A. Aglagane leg.; sweep net.
Det: Achik Dorchin
Global distribution: West-Palearctic
Regional distribution: Fès-Meknès; Rabat-Salé-Kénitra; **Marrakech-Safi**; **Tanger-Tétouan-Al Hoceima**

***Eucera obscura* Smith, 1879**
• 2♀; Bouizakarn; 29.2003, −9.7425; 22 March 2023; alt. 837 m; A. Aglagane leg.; sweep net.
Det: Achik Dorchin
Global distribution: West-Palearctic
Regional distribution: Fès-Meknès; Marrakech-Safi; Drâa-Tafilalet; **Guelmim-Oued Noun**

***Eucera pumila* Klug, 1845**
• 1♀; Mejjat; 31.4804, −8.4185; 13 March 2023; alt. 516 m; A. Aglagane leg.; blue pan trap.
Det: Achik Dorchin
Global distribution: West-Palearctic
Regional distribution: Drâa-Tafilalet; **Marrakech-Safi**

***Eucera strigata* Lepeletier, 1841**
• 6♀, 4♂; Moulay-Bousselham; 34.8856, −6.2581; 24 May 2022; alt. −2 m; A. Skaou and A. Aglagane leg.; sweep net.
Det: Achik Dorchin
Global distribution: West-Palearctic
Regional distribution: Tanger-Tetouan-Al Hoceima; Oriental; Fès-Meknès; Marrakech-Safi; **Rabat-Salé-Kénitra**

***Eucera tricincta* Erichson, 1835**
• 1♀; Tiztoutine; 34.9395, −3.0978; 27 May 2022; alt. 230 m; A. Skaou leg.; sweep net.
Det: Achik Dorchin
Global distribution: Palearctic
Regional distribution: Fès-Meknès; Marrakech-Safi; Drâa-Tafilalet; **Oriental**

***Eucera vidua* Lepeletier, 1841**
• 2♂; Douar bou Azza; 31.5036, −7.9358; 22 March 2022; alt. 600 m; A. Skaou leg.; sweep net.
• 3♂; Mejjat; 31.4804, −8.4185; 20 February 2023; alt. 516 m; A. Skaou, A. Aglagane, and O. Er-rguibi leg.; sweep net. • 9♂; Mejjat; 31.4804, −8.4185; 6 March 2023; alt. 516 m; A. Aglagane and O. Er-rguibi leg.; sweep net. • 2♀, 9♂; Mejjat; 31.4804, −8.4185; 13 March 2023; alt. 516 m; A. Skaou, A. Aglagane, and O. Er-rguibi leg.; sweep net. • 1♂; Mejjat; 31.4804, −8.4185; 14 March 2023; alt. 516 m; A. Skaou leg.; sweep net. • 1♂; Ouaoutelt; 29.2003, −9.7425; 22 March 2023; alt. 480 m; A. Aglagane leg.; sweep net. • 1♂; Oukaïmeden; 31.2136, −7.8509; 10 June 2022; alt. 2650 m; A. Skaou leg.; sweep net.
Det: Achik Dorchin
Global distribution: Near endemic (Morocco, Algeria, Spain)
Regional distribution: Souss-Massa; Rabat-Salé-Kénitra; Drâa-Tafilalet; **Marrakech-Safi**; **Guelmim-Oued Noun**

***Eucera warnckei* Risch, 1999**
• 9♀; Boujdour; 25.9853, −14.4963; 25 March 2023; alt. 60 m; O. Er-rguibi leg.; sweep net.
Det: Achik Dorchin
Global distribution: Near endemic (Morocco, Tunisia)
Regional distribution: Drâa-Tafilalet; Souss-Massa; Guelmim-Oued Noun; **Laâyoune-Boujdour-Sakia El Hamra**

**Family Colletidae Lepeletier, 1841**
**Genus *Hylaeus* Fabricius, 1793**

***Hylaeus clypearis* Schenck, 1853**
• 1♀; Bouizakarn; 29.2003, −9.7425; 22 March 2023; alt. 837 m; A. Aglagane leg.; sweep net.
Det: Romain Le Divelec
Global distribution: West-Palearctic
Regional distribution: Fès-Meknès; Marrakech-Safi; **Guelmim-Oued Noun**

**Family Halictidae Thomson, 1869**
**Genus *Ceylalictus* Strand, 1913**

***Ceylalictus variegatus* Olivier, 1789**
• 3♀; Laayoune; 27.0301, −13.0950; 23 March 2023; alt. 89 m; O. Er-rguibi leg.; sweep net.
Det: Thomas Brau
Global distribution: Palearctic; Sub-Saharan Africa
Regional distribution: Tanger-Tetouan-Al Hoceima; Oriental; Fès-Meknès; Rabat-Salé-Kénitra; Marrakech-Safi; Drâa-Tafilalet; Souss-Massa; Guelmim-Oued Noun; **Laâyoune-Boujdour-Sakia El Hamra**

**Genus *Lasioglossum* Curtis, 1833**

***Lasioglossum algericolellum* Strand, 1909**
• 3♀; Asni; 31.2508, −7.9855; 1 April 2022; alt. 1225 m; A. Skaou leg.; sweep net. • 1♀; Asni; 31.2534, −7.9781; 2 April 2024; alt. 1155 m; A. Skaou leg.; yellow pan trap. • 11♀; Asni; 31.2534, −7.9781; 6 April 2024; alt. 1155 m; A. Skaou, A. Chourou, and H. Benkhalifa leg.; yellow pan trap and sweep net. • 7♀, 1♂; Asni; 31.2534, −7.9781; 09 May 2024; alt. 1155 m; A. Skaou, A. Chourou, and H. Benkhalifa leg.; yellow pan traps and sweep net. • 14♀; Asni; 31.2534, −7.9781; 16 May 2024; alt. 1155 m; A. Skaou, A. Chourou, and H. Benkhalifa leg.; yellow, blue and white pan traps and sweep net. • 1♀, 1♂; Asni; 31.2534, −7.9781; 20 May 2024; alt. 1155 m; A. Skaou leg.; sweep net. • 37♀; Asni; 31.2534, −7.9781; 24 May 2024; alt. 1155 m; A. Skaou and H. Benkhalifa leg.; yellow, blue and white pan traps and sweep net. • 16♀; Asni; 31.2534, −7.9781; 25 May 2024; alt. 1155 m; A. Skaou and H. Benkhalifa leg.; sweep net. • 1♀; Mejjat; 31.4804, −8.4185; 20 February 2023; alt. 516 m; O. Er-rguibi leg.; blue pan trap. • 1♀; Mejjat; 31.4804, −8.4185; 13 March 2023; alt. 516 m; A. Skaou leg.; yellow pan trap. • 1♀; Moulay Brahim; 31.2793, −7.9601; 28 May 2024; alt. 1075 m; A. Skaou leg.; sweep net. • 1♀, 1♂; Moulay Brahim; 31.2793, −7.9601; 1 June 2024; alt. 1075 m; A. Skaou leg.; sweep net. • 3♀; Oukaïmeden; 31.2136, −7.8509; 9 May 2023; alt. 2650 m; A. Skaou leg.; sweep net. • 1♀, 2♂; Tikhfist; 31.2558, −7.8191; 8 May 2023; alt. 1780 m; A. Skaou leg.; sweep net. • 1♂, 3 sex unknown; Tikhfist; 31.2558, −7.8191; 7 June 2024; alt. 1780 m; A. Skaou leg.; sweep net.
Det: Simon Flaminio
Global distribution: West-Palearctic
Regional distribution: Tanger-Tetouan-Al Hoceima; Oriental; Fès-Meknès; Rabat-Salé-Kénitra; Béni Mellal-Khénifra; Casablanca-Settat; **Marrakech-Safi**

***Lasioglossum cristula* Pérez, 1896**
• 1♀; Fnideq; 35.8763, −5.3941; 25 May 2022; alt. 400 m; A. Skaou leg.; sweep net.
Det: Simon Flaminio
Global distribution: Palearctic
Regional distribution: Fès-Meknès; Rabat-Salé-Kénitra; **Tanger-Tetouan-Al Hoceima**

***Lasioglossum interruptum* Panzer, 1798**
• 1♀; Asni; 31.2508, −7.9855; 12 March 2024; alt. 1240 m; A. Aglagane leg.; sweep net. • 2♀; Asni; 31.2508, −7.9855; 2 April 2024; alt. 1155 m; A. Skaou, A. Aglagane leg.; blue and white traps. • 3♀; Asni; 31.2508, −7.9855; 6 April 2024; alt. 1155 m; A. Skaou, A. Aglagane leg.; white pan trap and sweep net. • 1♀; Asni; 31.2508, −7.9855; 16 April 2024; alt. 1155 m; A. Skaou leg.; blue pan trap and sweep net. • 1♂; Asni; 31.2508, −7.9855; 9 May 2024; alt. 1155 m; A. Skaou leg.; yellow pan trap. • 1♀; Asni; 31.2508, −7.9855; 16 May 2024; alt. 1155 m; A. Skaou leg.; yellow pan trap. • 2♀; Mejjat; 31.4804, −8.4185; 6 March 2023; alt. 516 m; A. Skaou and O. Er-rguibi leg.; sweep net. • 1♀; Mejjat; 31.4804, −8.4185; 13 March 2023; alt. 516 m; O. Er-rguibi leg.; sweep net. • 1♀; Moulay Brahim; 31.2793, −7.9601; 25 May 2024; alt. 1075 m; A. Skaou leg.; sweep net. • ♂♀; Moulay Brahim; 31.2793, −7.9601; 28 May 2024; alt. 1075 m; A. Skaou leg.; sweep net. • 2♂; Oukaïmeden; 31.2136, −7.8509; 9 May 2023; alt. 2650 m; A. Skaou leg.; sweep net. • 2 sex unknown; Safi; 32.3168, −9.2038; 8 June 2024; alt. 125 m; A. Skaou and Y. Dbiba leg.; sweep net. • 2♀; Tafetachte; 31.5907, −9.1878; 24 April 2024; alt. 423 m; A. Skaou leg.; sweep net. • 14♀; Tikhfist; 31.2558, −7.8191; 7 April 2024; alt. 1780 m; A. Skaou leg.; sweep net.
Det: Simon Flaminio
Global distribution: Nearctic and Palearctic
Regional distribution: Casablanca-Settat; Drâa-Tafilalet; Souss-Massa; **Marrakech-Safi**

***Lasioglossum lucidulum* Schenck, 1861**
• 1♀; Asni; 31.2534, −7.9781; 2 April 2024; alt. 1155 m; A. Skaou, A. Aglagane leg.; white pan trap. • 1♀; Asni; 31.2534, −7.9781; 30 April 2024; alt. 1155 m; A. Aglagane leg.; yellow pan trap. • 1♀; Asni; 31.2534, −7.9781; 16 May 2024; alt. 1155 m; A. Skaou leg.; yellow pan trap. • 1♀; Mejjat; 31.4804, −8.4185; 30 May 2023; alt. 516 m; A. Skaou leg.; sweep net. • 4♀; Moulay Brahim; 31.2793, −7.9601; 28 May 2024; alt. 1075 m; A. Skaou leg.; sweep net.
Det: Simon Flaminio
Global distribution: Palearctic
Regional distribution: Drâa-Tafilalet; Souss-Massa; **Marrakech-Safi**

***Lasioglossum malachurum* Kirby 1802**
• 4♀; Asni; 31.2615, −7.9450; 12 March 2024; alt. 1240 m; A. Aglagane, A. Chourou and H. Benkhalifa leg.; sweep net. • 1♀; Asni; 31.2534, −7.9781; 2 April 2024; alt. 1155 m; A. Skaou leg.; yellow pan trap. • 1♀; Asni; 31.2534, −7.9781; 30 April 2024; alt. 1155 m; A. Skaou leg.; white pan trap. • 2♀; Asni; 31.2615, −7.9450; 24 May 2024; alt. 1240 m; A. Skaou and H. Benkhalifa leg.; white pan trap and sweep net. • 1♀; Douar bou Azza; 31.5036, −7.9358; 22 March 2022; alt. 600 m; A. Skaou leg.; sweep net. • 1 sex unknown; Imlil; 31.1390, −7.9211; 10 May 2024; alt. 1758 m; A. Skaou leg.; sweep net. • 1♀; Oukaïmeden; 31.2136, −7.8509; 21 February 2023; alt. 2650 m; A. Skaou leg.; sweep net. • 2♀; Tighdouine; 31.4144, −7.5297; 21 March 2022; alt. 1094 m; A. Skaou and A. Aglagane leg.; Pan trap (color unknown). • 3♀; Tikhfist; 31.2558, −7.8191; 12 June 2023; alt. 1765 m; A. Skaou leg.; sweep net.
Det: Simon Flaminio
Global distribution: Palearctic
Regional distribution: Tanger-Tetouan-Al Hoceima; Fès-Meknès; Rabat-Salé-Kénitra; Souss-Massa; **Marrakech-Safi**

***Lasioglossum minutissimum* Kirby, 1802**
• 3♀; Asni; 31.2534, −7.9781; 2 April 2024; alt. 1155 m; A. Skaou and A. Aglagane leg.; blue, yellow, and white pan traps. • 2♀; Asni; 31.2534, −7.9781; 30 April 2024; alt. 1155 m; A. Skaou and A. Aglagane leg.; blue and yellow pan traps. • 2♀; Asni; 31.2534, −7.9781; 9 May 2024; alt. 1155 m; A. Skaou leg.; yellow, and white pan trap. • 4♀; Asni; 31.2534, −7.9781; 16 May 2024; alt. 1155 m; A. Skaou, A. Chourou and H. Benkhalifa leg.; blue and white pan traps. • 8♀; Asni; 31.2534, −7.9781; 24 May 2024; alt. 1155 m; A. Skaou, A. Chourou, and H. Benkhalifa leg.; blue, yellow, and white pan traps. • 1♀; Asni; 31.2534, −7.9781; 25 May 2024; alt. 1155 m; A. Skaou leg.; sweep net. • 3♀; Mejjat; 31.4804, −8.4185; 20 February 2023; alt. 516 m; A. Aglagane and O. Er-rguibi leg.; blue and white pan traps. • 1♀; Mejjat; 31.4804, −8.4185; 6 March 2023; alt. 516 m; A. Skaou leg.; sweep net. • 1♀; Moulay Brahim; 31.2793, −7.9601; 28 May 2024; alt. 1758 m; A. Skaou leg.; sweep net. • 1♀; Moulay Brahim; 31.2793, −7.9601; 1 June 2024; alt. 1758 m; A. Skaou leg.; sweep net.
Det: Simon Flaminio
Global distribution: Palearctic
Regional distribution: Fès-Meknès; Drâa-Tafilalet; Souss-Massa; **Marrakech-Safi**

***Lasioglossum parvulum* Schenck 1853**
• 1♀; Imlil; 31.1390, −7.9211; 10 May 2024; alt. 1758 m; A. Skaou leg.; sweep net.
Det: Simon Flaminio
Global distribution: West-Palearctic
Regional distribution: **Marrakech-Safi**

***Lasioglossum pauliani* Benoist, 1941**
• 1♂; Moulay Brahim; 31.2793, −7.9601; 1 June 2024; alt. 1075 m; A. Skaou leg.; sweep net.
Det: Simon Flaminio
Global distribution: Morocco. ENDEMIC
Regional distribution: Fès-Meknès; **Marrakech-Safi**

***Lasioglossum pauxillum* Schenck, 1853**
• 1♀; Asni; 31.2534, −7.9781; 9 May 2024; alt. 516 m; A. Skaou leg.; sweep net.
Det: Simon Flaminio
Global distribution: Palearctic
Regional distribution: Tanger-Tetouan-Al Hoceima; Oriental; Fès-Meknès; Rabat-Salé-Kénitra; Béni Mellal-Khénifra; Casablanca-Settat; **Marrakech-Safi**

***Lasioglossum xanthopus* Kirby, 1802**
• 1♀; Oukaïmeden; 31.2136, −7.8509; 10 June 2022; alt. 2650 m; A. Skaou leg.; sweep net.
Det: Simon Flaminio
Global distribution: Palearctic
Regional distribution: Rabat-Salé-Kénitra; Tanger-Tétouan-Al Hoceima; **Marrakech-Safi**

**Genus *Nomiapis* Cockerell, 1919**

***Nomiapis rufiventris* Spinola, 1838**
• 1♀; Chichaoua; 31.5498, −8.8033; 24 April 2024; alt. 420 m; A. Skaou leg.; sweep net. • 1♀; Echemmaia; 32.0930, −8.6560; 8 April 2024; alt. 410 m; A. Skaou leg.; sweep net. • 1♀; Marrakech; 31.6122, −8.0050; 15 May 2022; alt. 479 m; A. Skaou leg.; sweep net. • 2♀, 1♂; Marrakech; 31.6097, −7.9897; 31 May 2022; alt. 479 m; A. Skaou leg.; sweep net. • 3♀; Mejjat; 31.4804, −8.4185; 30 May 2023; alt. 516 m; A. Skaou leg.; sweep net. • 1 sex unknown; Mejjat; 31.5465, −9.3774; 24 April 2024; alt. 413 m; A. Skaou leg.; sweep net. • 2♀; Oued Tensift; 31.7091, −8.0738; 1 June 2022; alt. 378 m; A. Skaou leg.; sweep net. • 1♀; Oued Tensift; 31.7091, −8.0738; 7 Jun 2022; alt. 378 m; A. Skaou leg.; sweep net. • 1♀; Tikhfist; 31.2558, −7.8191; 7 June 2024; alt. 1780 m; A. Skaou leg.; sweep net. • 2♂; Timzilite; 31.5385, −7.5720; 9 May 2022; alt. 806 m; A. Skaou and A. Aglagane leg.; sweep net.
Det: Simone Flaminio
Global distribution: West-Palearctic
Regional distribution: Souss-Massa; **Marrakech-Safi**

**Genus *Pseudapis* Kirby, 1900**

***Pseudapis nilotica* Smith, 1875**
• 2♀; Laayoune; 27.0301, −13.0950; 23 March 2023; alt. 89 m; A. Aglagane and O. Er-rguibi leg.; sweep net.
Det: Simone Flaminio
Global distribution: Palearctic; Sub-Saharan Africa
Regional distribution: Oriental; Béni Mellal-Khénifra; Casablanca-Settat; Marrakech-Safi; Drâa-Tafilalet; Souss-Massa; Guelmim-Oued Noun; Dakhla-Oued ed-Dahab; **Laâyoune-Boujdour-Sakia El Hamra**

**Genus *Seladonia* Robertson, 1918**

***Seladonia cupida* Vachal, 1902**
• 1 sex unknown; Laayoune; 27.1577, −13.2293; 23 March 2023; alt. 89 m; A. Aglagane leg.; sweep net.
Det: Thomas Brau
Global distribution: Palearctic
Regional distribution: Oriental; Drâa-Tafilalet; Souss-Massa; **Laâyoune-Boujdour-Sakia El Hamra**

***Seladonia ochropa* Blüthgen, 1923**
• 1 sex unknown; Boujdour; 25.9853, −14.4963; 25 March 2023; alt. 60 m; A. Aglagane leg.; sweep net.
Det: Thomas Brau
Global distribution: Near endemic (Morocco, Algeria)
Regional distribution: Drâa-Tafilalet; Fès-Meknès; Oriental; Souss-Massa; **Laâyoune-Boujdour-Sakia El Hamra**

***Seladonia subaurata* Rossi, 1792**
• 1 sex unknown; Mejjat; 31.4804, −8.4185; 13 March 2023; alt. 516 m; A. Skaou leg.; yellow pan trap.
Det: Thomas Brau
Global distribution: Palearctic
Regional distribution: Fès-Meknès; Rabat-Salé-Kénitra; Drâa-Tafilalet; **Marrakech-Safi**

**Genus *Sphecodes* Latreille, 1804**

***Sphecodes rubicundus* Hagens, 1875**
• 1♀; Amizmiz; 31.2183, −8.2416; 13 April 2022; alt. 965 m; A. Skaou leg.; sweep net. • 1♀, 1♂; Mejjat; 31.4804, −8.4185; 13 March 2023; alt. 516 m; A. Skaou and O. Er-rguibi leg.; sweep net.
Det: Jakub Straka
Global distribution: West-Palearctic
Regional distribution: Tanger-Tetouan-Al Hoceima; **Marrakech-Safi**

**Family Megachilidae Latreille, 1802**
**Genus *Anthidium* Fabricius, 1804**

***Anthidium cingulatum* Latreille, 1809**
• 1♂; Ksar Sghir; 35.8234, −5.5370; 25 May 2022; alt. 132 m; A. Skaou leg.; sweep net.
Det: Max Kasparek
Global distribution: Palearctic
Regional distribution: Fès-Meknès; rabat-Salé-kénitra; Marrakech-Safi; Drâa-Tafilalet; Souss-Massa; **Tanger-Tétouan-Al Hoceima**

***Anthidium pullatum* Morice, 1916**
• 1♂; Ouaaroun; 28.9109, −10.1572; 23 March 2023; alt. 22 m; A. Aglagane leg.; sweep net.
Det: Max Kasparek
Global distribution: Near endemic (Morocco, Algeria)
Regional distribution: Drâa-Tafilalet; Souss-Massa; **Guelmim-Oued Noun**

**Genus *Haetosmia* Popov, 1952**

***Haetosmia circumventa* Peters, 1974**
• 1♀; Laayoune; 27.0301, −13.0950; 23 March 2023; alt. 89 m; O. Er-rguibi leg.; sweep net.
Det: Andreas Mueller
Global distribution: West-Palearctic; Sub-Saharan Africa
Regional distribution: Drâa-Tafilalet; Souss-Massa; Guelmim-Oued Noun; **Laâyoune-Boujdour-Sakia El Hamra**

**Genus *Hoplitis* Klug, 1807**

***Hoplitis hierichonica* Mavromoustakis, 1949**
• 2♂; Mejjat; 31.4804, −8.4185; 13 February 2023; alt. 516 m; A. Skaou leg.; sweep net • 1♂; Mejjat; 31.4804, −8.4185; 06 March 2023; alt. 516 m; O. Er-rguibi leg.; sweep net.
Det: Andreas Mueller
Global distribution: West-Palearctic
Regional distribution: Drâa-Tafilalet; Souss-Massa; Guelmim-Oued Noun; **Marrakech-Safi**

***Hoplitis mucida* Dours, 1873**
• 3♀; Al Hoceima; 35.2691, −3.6752; 27 May 2022; alt. 41 m; A. Skaou and A. Aglagane leg.; sweep net.
Det: Andreas Mueller
Global distribution: West-Palearctic
Regional distribution: Oriental; Fès-Meknès; Béni Mellal-Khénifra; Casablanca-Settat; Marrakech-Safi; Drâa-Tafilalet; Souss-Massa; **Tanger-Tétouan-Al Hoceima**

**Genus *Icteranthidium* Michener, 1948**

***Icteranthidium ferrugineum* Fabricius, 1787**
• 1♂; Boujdour; 25.9853, −14.4963; 25 March 2023; alt. 60 m; O. Er-rguibi leg.; sweep net.
Det: Max Kasparek
Global distribution: Palearctic; Sub-Saharan Africa
Regional distribution: Tanger-Tetouan-Al Hoceima; Oriental; Rabat-Salé-Kénitra; Marrakech-Safi; Drâa-Tafilalet; Souss-Massa; Guelmim-Oued Noun; **Laâyoune-Boujdour-Sakia El Hamra**

**Genus *Osmia* Panzer, 1806**

***Osmia latreillei* Spinola, 1806**
• 1♂; Boujdour; 25.9853, −14.4963; 25 March 2023; alt. 60 m; O. Er-rguibi leg.; sweep net.
Det: Andreas Mueller
Global distribution: West-Palearctic
Regional distribution: Tanger-Tetouan-Al Hoceima; Oriental; Fès-Meknès; Rabat-Salé-Kénitra; Béni Mellal-Khénifra; Marrakech-Safi; Drâa-Tafilalet; Souss-Massa; Guelmim-Oued Noun; **Laâyoune-Boujdour-Sakia El Hamra**

***Osmia leaiana* Kirby, 1802**
• 1♀; Tamernout; 35.5165, −5.1641; 26 May 2022; alt. 99 m; A. Skaou leg.; sweep net.
Det: Andreas Mueller
Global distribution: Palearctic
Regional distribution: Fès-Meknès; Marrakech-Safi; Drâa-Tafilalet; **Tanger-Tetouan-Al Hoceima**

***Osmia melanogaster* Spinola, 1808**
• 1♀; Tighdouine; 31.5385, −7.5720; 9 June 2022; alt. 806 m; A. Skaou leg.; sweep net.
Det: Andreas Mueller
Global distribution: Palearctic
Regional distribution: Tanger-Tetouan-Al Hoceima; Oriental; Fès-Meknès; Drâa-Tafilalet; **Marrakech-Safi**

**Family Melittidae Schenck, 1860**
**Genus *Melitta* Kirby, 1802**

***Melitta schmiedeknechti* Friese, 1898**
• 3♂; Boujdour; 25.9853, −14.4963; 25 March 2023; alt. 60 m; O. Er-rguibi and A. Aglagane leg.; sweep net.
Det: Denis Michez
Global distribution: West-Palearctic
Regional distribution: Béni Mellal-Khénifra; Drâa-Tafilalet; Fès-Meknès; Guelmim-Oued Noun; Marrakech-Safi; Oriental; Rabat-Salé-Kénitra; Souss-Massa; Tanger-Tétouan-Al Hoceima; **Laâyoune-Boujdour-Sakia El Hamra**
